# Preferential antitumor effect of the Src inhibitor dasatinib associated with a decreased proportion of aldehyde dehydrogenase 1-positive cells in breast cancer cells of the basal B subtype

**DOI:** 10.1186/1471-2407-10-568

**Published:** 2010-10-20

**Authors:** Junichi Kurebayashi, Naoki Kanomata, Takuya Moriya, Yuji Kozuka, Mika Watanabe, Hiroshi Sonoo

**Affiliations:** 1 Department of Breast and Thyroid Surgery, Kawasaki Medical School, 577 Matsushima, Kurashiki, Okayama 701-0192, Japan; 2 Department of Pathology, Kawasaki Medical School, 577 Matsushima, Kurashiki, Okayama 701-0192, Japan; 3 Department of Pathology, Tohoku University Hospital, 1-1 Seiryo-machi, Aoba-ku, Sendai 980-8574, Japan

## Abstract

**Background:**

Recent studies have suggested that the Src inhibitor dasatinib preferentially inhibits the growth of breast cancer cells of the basal-like subtype. To clarify this finding and further investigate combined antitumor effects of dasatinib with cytotoxic agents, a panel of breast cancer cell lines of various subtypes was treated with dasatinib and/or chemotherapeutic agents.

**Methods:**

Seven human breast cancer cell lines were treated with dasatinib and/or seven chemotherapeutic agents. Effects of the treatments on c-Src activation, cell growth, cell cycle, apoptosis and the proportion of aldehyde dehydrogenase (ALDH) 1-positive cells were examined.

**Results:**

The 50%-growth inhibitory concentrations (IC_50_s) of dasatinib were much lower in two basal B cell lines than those in the other cell lines. The IC_50_s of chemotherapeutic agents were not substantially different among the cell lines. Dasatinib enhanced antitumor activity of etoposide in the basal B cell lines. Dasatinib induced a G1-S blockade with a slight apoptosis, and a combined treatment of dasatinib with etoposide also induced a G1-S blockade in the basal B cell lines. Dasatinib decreased the expression levels of phosphorylated Src in all cell lines. Interestingly, dasatinib significantly decreased the proportion of ALDH1-positive cells in the basal B cell lines but not in the other cell lines.

**Conclusions:**

The present study indicates that dasatinib preferentially inhibits the growth of breast cancer cells of the basal B subtype associated with a significant loss of putative cancer stem cell population. A combined use of dasatinib with etoposide additively inhibits their growth. Further studies targeting breast cancers of the basal B subtype using dasatinib with cytotoxic agents are warranted.

## Background

Gene expression microarray analyses have revealed that breast cancers consist of several distinctive subtypes [1,2]. Most breast cancers classified into the basal-like subtype have an estrogen receptor (ER)-negative, progesterone receptor (PgR)-negative and human epidermal growth factor receptor (HER) 2-negative (so-called "triple-negative") phenotype [3]. Because of the lack of molecular targets in triple-negative/basal-like breast cancers and their aggressive biological behaviors, novel treatment strategies against them have been intensively investigated [4].

Recent preclinical studies have shown that a multiple tyrosine kinase inhibitor, dasatinib, has a more potent antitumor effect on triple negative/basal-like breast cancer cells than those of other subtypes [5,6]. It is suggested that one of the targets of dasatinib, the Src tyrosine kinase pathway, is responsible for its antitumor activity. Otherwise, several molecular markers for predicting the antitumor activity of dasatinib have been already reported [6].

A series of preclinical and clinical studies have indicated that most triple negative/basal-like breast cancers have dysfunctional BRCA1 or loss of BRCA1 expression [7-9]. BRCA1 plays important roles in maintenance of genetic stability including DNA double-strand break repair [10]. Preclinical and clinical studies have suggested that triple negative/basal-like breast cancers are sensitive to DNA-damaging agents such as cisplatin (Cis) [10,11].

To clarify preferential antitumor activity of dasatinib and DNA-damaging agents in triple negative/basal-like breast cancer cells, we investigated antitumor effects of dasatinib and various chemotherapeutic agents including DNA-damaging agents on a panel of breast cancer cell lines of various subtypes. In addition, in consideration of future clinical implications, combined antitumor activity of dasatinib with cytotoxic agents was also investigated. Furthermore, because recent translational studies have suggested that molecular targeting agents such as trastuzumab and lapatinib may effectively decrease the proportion of breast cancer stem cells associated with a significant inhibition of non-stem cell growth, changes in the proportion of aldehyde dehydrogenase (ALDH) 1-positive cells, which may represent cancer stem cells, were also examined [12-14].

## Methods

### Reagents

Dasatinib was provided by Bristol-Myers Squibb Pharmaceutical Research Institute (Princeton, NJ). Etoposide (Eto), doxorubicin (Dox), 5-fluorouracil (FU), paclitaxel (Pac), Cis and carboplatin (Carb) were purchased from Sigma Co. (St Louis, MI). An active metabolite of irinotecan hydrochloride, SN38 was provided by Dai-ichi Sankyo Pharmaceutical Co. (Tokyo, Japan).

### Breast cancer cell Lines and culture conditions

The KPL-1, KPL-3C and KPL-4 breast cancer cell lines were established in our laboratory [15-17]. The MDA-MB-231 cell line was provided by late Dr. Robert B. Dickson (Lombardi Cancer Research Center, Georgetown University Medical Center, Washington DC). The MDA-MB-157, BT-474 and HCC-1937 cell lines were obtained from the American Type Culture Collection (Rockville, MD). All cell lines were routinely maintained in Dulbecco's modified Eagle's medium (D-MEM) supplemented with 10% fetal bovine serum (FBS).

### Cell growth analysis

To investigate the effects of dasatinib and/or chemotherapeutic agents on cell growth, breast cancer cells (1-5 × 10^4 ^cells per well) were seeded in 24-well plates (SB Medical, Tokyo, Japan) and grown in D-MEM supplemented with 10% FBS at 37°C in a 5% CO_2 _atmosphere for two days. After washing with phosphate-buffered saline (PBS), the cells were incubated with D-MEM supplemented with 10% FBS plus indicated concentrations of dasatinib and/or chemotherapeutic agents for three days. In the combination treatment, the cells were incubated with D-MEM supplemented with 10% FBS plus 0.1 *μ*M dasatinib and the indicated concentrations of the respective chemotherapeutic agents for three days. After the incubation, the cells were harvested and counted with a Coulter counter (Coulter Electronics, Harpenden, UK). Reproducibility was confirmed in at least two separate experiments.

To evaluate the antitumor effects of combined treatments, a combination index based on 50% inhibitory concentration (IC_50_) was calculated according to the following formula: combination index = IC_50 _with combined treatment/IC_50 _with single treatment. Combination index < 0.5 was considered evidence of additive interaction [18].

### Cell cycle and apoptosis analyses

To investigate the effect of agents on cell cycle progression, harvested cells were stained with propidium iodide using a CycleTest Plus DNA Reagent kit (Becton Dickinson, San Jose, CA). Flow cytometry was performed with a FACSCalibur flow cytometer (Becton Dickinson), and the DNA histogram was analyzed using CELLQuest version 1.2.2 (Becton Dickinson).

To investigate the effect of agents on apoptosis, approximately 5 × 10^5 ^cells per well were plated into T-25 flasks (Corning Japan, Tokyo, Japan) and cultured in D-MEM supplemented with 10% FBS for two days. The cells were then washed twice with PBS and cultured for two days in D-MEM supplemented with 10% FBS plus the indicated concentrations of dasatinib and/or chemotherapeutic agents. Duplicate flasks were trypsinized and harvested. The percentages of apoptotic cells were measured by FACSCalibur flow cytometry (Becton Dickinson) using an Annexin-V-FLUOS staining kit (Roche Diagnostics GmbH, Penzberg, Germany) according to the manufacturer's recommendations.

### Immunocytochemistry

Harvested cells were washed once with cold PBS and centrifuged at room temperature. The cell pellet was fixed with 10% phosphate-buffered formalin overnight and embedded in paraffin. The 5 *μ*m paraffin sections were dewaxed with xylene, hydrated with PBS, treated with hydrogen peroxide and processed following an immunoperoxidase procedure. The primary antibodies used were: ER-α (1D5, monoclonal, 1:400, IMMUNOTECH, Marseilles, France), PgR (PgR636, monoclonal, 1:2,000, DAKO, Copenhagen, Denmark), HER2 (HercepTest II, DAKO), HER1 (2-18C9, monoclonal, DAKO), cytokeratin 5/6 (D5/16B4, monoclonal, 1:100, DAKO), BRCA1 (GLR-2, monoclonal, 1:100, DAKO), p53 (DO-7, monoclonal, 1:100, Nichirei Bioscience, Tokyo, Japan), vimentin (V9, monoclonal, 1:100, DAKO), c-Src (Src antibody, polyclonal, 1:800, Cell Signaling Technology, Danvers, MA), p-SrcY416 (phosphor-Src family Tyr416 antibody, polyclonal, 1:100; Cell Signaling Technology), p-Src Y527 (phosphor-Src family Tyr527 antibody; polyclonal, 1:50; Cell Signaling Technology), and ALDH1 (monoclonal, IG isotype, 1:100; BD Biosciences, San Jose, CA). After incubation, the slides were washed in PBS or Tris-buffered saline for 15 min, and each secondary antibody (mouse or rabbit) was applied, using the LSAB kit (DAKO) or using the EnVision kit (DAKO). After washing, the color was developed with 5-bromo-4-chloro-3-indoxyl phosphate and nitroblue tetrazolium chloride (DAKO). The slides were then washed in distilled water for 5 min and mounted. Control experiments were performed by substituting normal rabbit or mouse serum for the first antibody.

### Aldefluor assay

The ALDEFLUOR kit (StemCell Technologies, Durham, NC) was used to isolate the population with a high ALDH enzymatic activity. Harvested cells were suspended in the ALDFLUOR assay buffer containing ALDH substrate (BAAA, 1 *μ*mol/l per 1 × 10^6 ^cells) and incubated for 40 minutes at room temperature. As negative control, cells were treated with 50-mmol/l diethylaminobenzaldehyde (DEAB), a specific ALDH inhibitor.

### Statistical analysis

All values are expressed as the mean ± SE. ANOVA analysis with StatView computer software (ATMS Co., Tokyo, Japan) was used to compare the differences between two groups. A two-sided *P *value less than 0.05 was considered statistically significant.

## Results

### Characteristics of breast cancer cell lines

To examine biological characteristics of a panel of breast cancer cell lines, immunocytochemical analyses for ER-α, PgR, HER2, HER1, cytokeratin 5/6, vimentin, BRCA1, p53, c-Src, p-Src (Y416) and p-Src (Y527) were performed (Additional file 1). According to the immunohistochemical intrinsic subtypes, the KPL-1 and KPL-3C cell lines were categorized as the luminal A subtype, the BT-474 cell line as the luminal B subtype, the KPL-4 cell line as the HER2-positive/ER-negative subtype, and the MDA-MB-231, MD-MB-157 and HCC1937 cell lines as the basal-like subtype [3]. HER1 overexpression was observed in cell lines of the basal-like subtype. No detectable expression of BRCA1 was observed in cell lines of the basal-like subtype. An active form of c-Src, p-Src (Y416) was overexpressed in cell lines of the basal-like subtype. Overexpression of vimentin was observed in the MDA-MB-231 and MD-MB-157 cell lines but not in the HCC1937 cell lines. According to these findings and others', the MDA-MB-231 and MD-MB-157 cell lines are sub-categorized as the basal B subtype, and the HCC1937 is as the basal A subtype [19].

### Effects of dasatinib on Src phosphorylation

**Figure 1:**
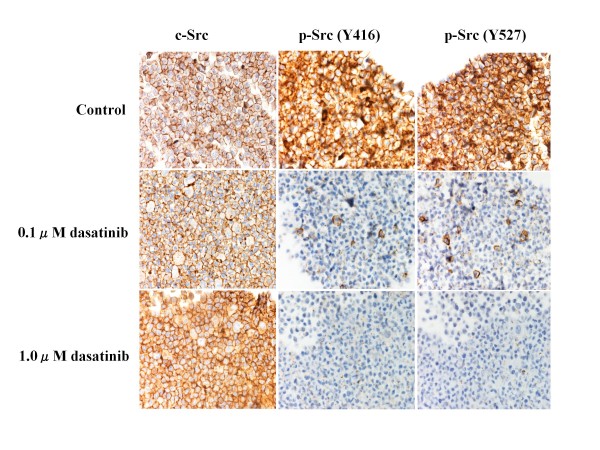
**Effects of dasatinib on c-Src phosphorylation in MDA-MB-231 cells**. Breast cancer cells were treated with indicated concentrations of dasatinib for 24 hours. The expression levels of c-Src, p-Src (Y416) and p-Src (Y527) were measured by the immunocytochemical analysis described in the Materials and Methods. Dasatinib dose-dependently decreased the expression levels of p-Src (Y416 and Y-527) but not that of c-Src.

**Figure 2:**
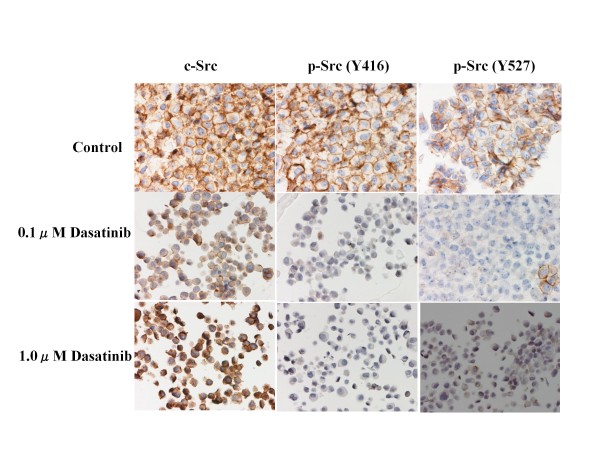
**Effects of dasatinib on c-Src phosphorylation in MDA-MB-157 cells**. **D**asatinib dose-dependently decreased the expression levels of p-Src (Y416 and Y-527) but not that of c-Src.

**Figure 3:**
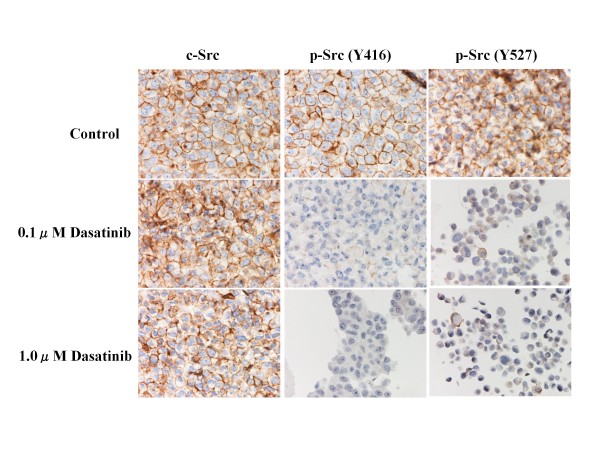
**Effects of dasatinib on c-Src phosphorylation in BT-474 cells**. Dasatinib dose-dependently decreased the expression levels of p-Src (Y416 and Y-527) but not that of c-Src.

To clarify anti-Src activity of dasatinib on breast cancer cells, changes in expression levels of c-Src and p-Src (Y416 and Y527) were examined before and after the treatment with dasatinib (0.1 or 1.0 *μ*M) for 24 hours. Although dasatinib did not significantly change the expression levels of c-Src in any of the cell lines, dasatinib significantly suppressed the expression levels of either p-Src (Y416) or p-Src (Y527) in all cell lines in a dose-dependent manner (Figures 1, 2 and 3).

### Antitumor activity of dasatinib and/or chemotherapeutic agents

The IC_50 _for dasatinib was much lower than the reported trough concentration of dasatinib in humans (0.6 *μ*M) in the MDA-MB-231 and MDA-MB-157 cell lines (0.15 *μ*M and 0.10 *μ*M, respectively) [6]. In contrast, that for dasatinib was equal or over than 5.0 *μ*M in the other cell lines tested (Additional file 2).

The IC_50_s for Eto, Dox, Pac, Cis and SN38 were lowest in the cell lines of basal B subtype but not those for 5-FU and Carb among the cell lines tested (Additional file 2). The IC_50_s of chemotherapeutic agents were not substantially different among the cell lines compared with dasatinib.

**Figure 4:**
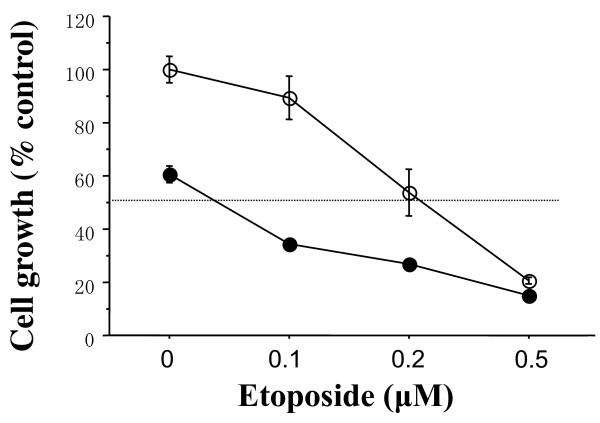
**Additive antitumor activity of dasatinib and Eto in MDA-MB-231 cells**. The cells were treated with indicated concentrations of Eto and/or 0.1 *μ*M dasatinib for three days, and the cell count was measured by a Coulter counter as described in the Materials and Methods. Dasatinib additively inhibited the growth of MDA-MB-231 cells. Open circles represent values treated with Eto alone. Closed circles represent values treated with Eto plus dasatinib. The values are expressed as mean ± S.E. of at least two separate experiments.

**Figure 5:**
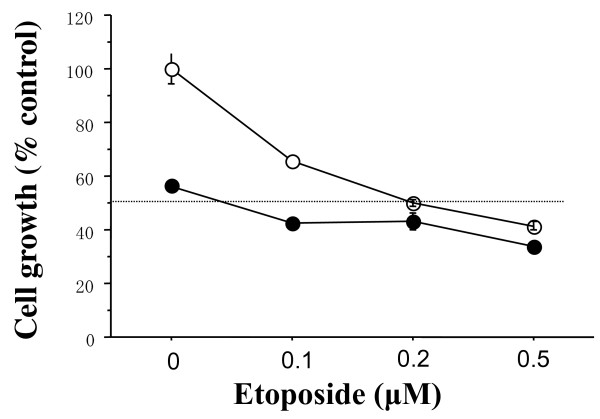
**Additive antitumor activity of dasatinib and Eto in MDA-MB-157 cells**. The cells were treated with indicated concentrations of Eto and/or 0.1 *μ*M dasatinib for three days. Dasatinib additively inhibited the growth of MDA-MB-157 cells. Open circles represent values treated with Eto alone. Closed circles represent values treated with Eto plus dasatinib. The values are expressed as mean ± S.E. of at least two separate experiments.

To explore combined antitumor effects of dasatinib with cytotoxic agents, combined treatments of dasatinib (0.1 *μ*M) with the indicated concentrations of Eto were examined in the basal B cell lines. The combination index of the IC_50 _was 0.2 in either cell line, that is, dasatinib additively enhanced the antitumor effect of Eto in these cell lines (Figures 4 and 5). Similar additive interactions were also observed in the combination of dasatinib with Pac or SN38 in these cell lines (Supplementary Figure 2). These additive interactions were not observed in the other cell lines.

**Figure 6:**
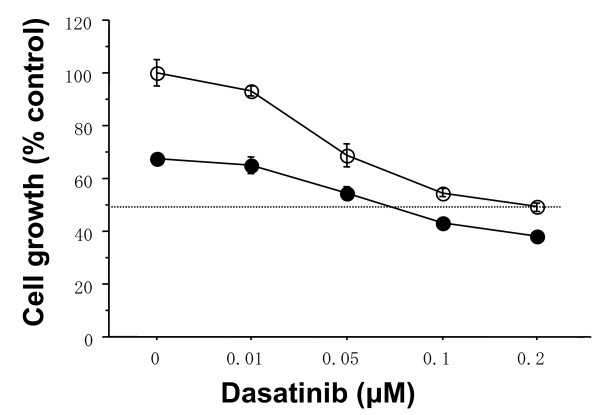
**Additive antitumor activity of dasatinib and Eto in MDA-MB-231 cells**. The cells were treated with indicated concentrations of dasatinib and/or 0.1 *μ*M Eto for three days. Eto additively inhibited the growth of MDA-MB-157 cells. Open circles represent values treated with dasatinib alone. Closed circles represent values treated with Eto plus dasatinib. The values are expressed as mean ± S.E. of at least two separate experiments.

**Figure 7:**
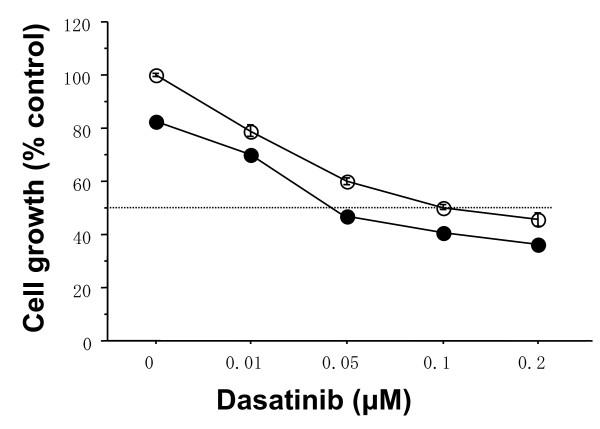
**Additive antitumor activity of dasatinib and Eto in MDA-MB-157 cells**. The cells were treated with indicated concentrations of dasatinib and/or 0.1 *μ*M Eto for three days. Eto additively inhibited the growth of MDA-MB-157 cells. Open circles represent values treated with dasatinib alone. Closed circles represent values treated with Eto plus dasatinib. The values are expressed as mean ± S.E. of at least two separate experiments.

To further clarify the combined antitumor effects of dasatinib and Eto, combined treatments with Eto (0.1 *μ*M) with the indicated concentrations of dasatinib were examined in the basal B cell lines. The combination index of IC_50 _was 0.3 or 0.4 in the MDA-MB-231 or MDA-MB-157 cell line, respectively, that is, Eto additively enhanced the antitumor effect of dasatinib in these cell lines (Figures 6 and 7).

### Effects of dasatinib and Eto on cell cycle progression and induction of apoptosis

**Figure 8:**
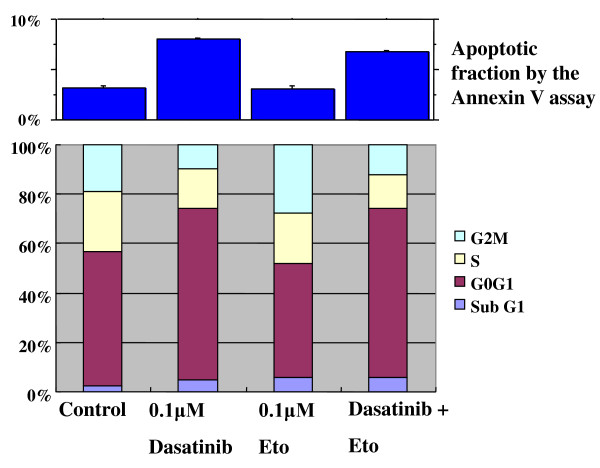
**Effects of dasatinib and Eto on cell cycle progression and induction of apoptosis in MDA-MB-231 cells**. The cells were treated with 0.1 *μ*M Eto and/or 0.1 *μ*M dasatinib for two days, and cell cycle and apoptosis were measured as described in the Materials and Methods. Dasatinib induced a G1-S blockade with a slight apoptosis, Eto induced a G2-M accumulation, and the combined treatment induced a G1-S blockade with a slight apoptosis in MDA-MB-231 cells.

**Figure 9:**
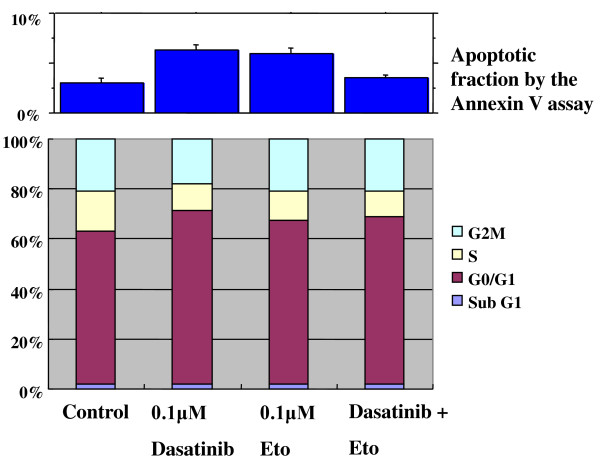
**Effects of dasatinib and Eto on cell cycle progression and induction of apoptosis in MDA-MB-157 cells**. Dasatinib induced a G1-S blockade with a slight apoptosis, Eto induced a slight G1-S blockade, and the combined treatment induced a G1-S blockade with a slight apoptosis in MDA-MB-157 cells.

To elucidate the mechanism of action of dasatinib and/or Eto in the cell lines of the basal B subtype, effects of single or combined treatments on cell cycle progression and induction of apoptosis were examined. Dasatinib (0.1 *μ*M) induced a significant G1-S cell cycle blockade associated with a slight apoptosis in both cell lines (Figures 8 and 9). Percentages of cells in the G0/G1 phase were 54.3 ± 0.2 for the control and 70.3 ± 0.9 for dasatinib (P < 0.01) in MDA-MB-231 cells. Those were 60.5 ± 0.3 for the control and 70.8 ± 1.4 for dasatinib (P < 0.01) in MDA-MB-157 cells. Percentages of apoptotic cells were 3.2 ± 0.2 for the control and 8.0 ± 0.1 for dasatinib (P < 0.01) in MDA-MB-231 cells. These were 3.0 ± 0.5 for the control and 6.3 ± 0.5 for dasatinib (P < 0.01) in MDA-MB-157 cells. Eto (0.1 *μ*M) induced a significant G2 accumulation in the MDA-MB-231 cell line (Figure 8). Percentages of cells in the G2/M phase were 18.8 ± 0.4 for the control and 25.4 ± 2.2 for Eto (P = 0.01) in MDA-MB-231 cells. Eto induced slight apoptosis in MDA-MB-157 cell lines (Figure 9). Percentages of apoptotic cells were 3.0 ± 0.5 for the control and 6.0 ± 0.6 for Eto (P = 0.01) in MDA-MB-157 cells.

A combined treatment with dasatinib (0.1 *μ*M) and Eto (0.1 *μ*M) induced a G1-S cell cycle blockade with or without a slight apoptosis in both cell lines (Figure 8 and 9). Percentages of cells in the G0/G1 phase were 69.6 ± 0.9 for combined treatment (P < 0.01 compared with the control) in MDA-MB-231 cells and 66.5 ± 0.7 for combined treatment (P = 0.01 compared with the control) in MDA-MB-157 cells. It should be noted that Eto did not show any additive effect to the treatment with dasatinib alone on G1-S cell cycle retardation and increased apoptosis in both cell lines. These findings suggest that the additive antitumor effect with dasatinib and Eto is unlikely to be due to enhanced cell cycle retardation or increased apoptosis in these cell lines.

### Effects of dasatinib and/or Eto on the proportion of ALDH1-positive cells

To test a hypothesis that dasatinib may more effectively kill breast cancer stem cells than chemotherapeutic agents, effects of dasatinib and/or Eto on the proportion of ALDH1-positive cells in a panel of breast cancer cell lines were examined using an immunocytochemical analysis and Aldefluor assay. The proportion of ALDH1-positive cells was higher than that by the Aldefluor assay but was in line with the results of the immunocytochemical assay. Base-line proportions of ALDH1-positive cells were higher in HER2-overexpressing BT-474 and KPL-4 cell lines than HER2-negative cell lines. Those of the cell lines of the luminal A subtype were less than 1% (Additional file 3).

**Figure 10:**
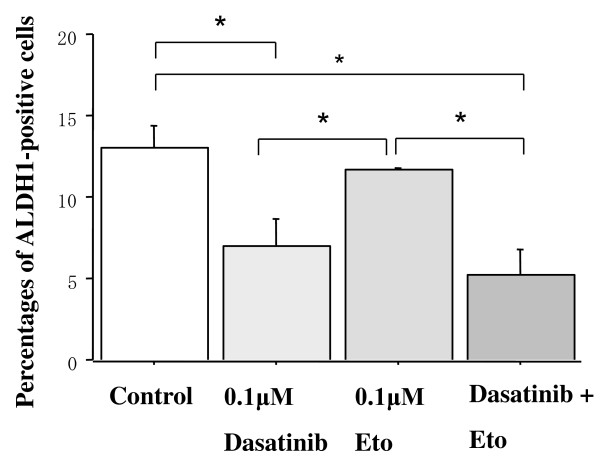
**Effects of dasatinib and/or Eto on the proportion of ALDH1-positive cells in MDA-MB-231 cells**. The cells were treated with 0.1 *μ*M Eto and/or 0.1 *μ*M dasatinib for two days, and ALDH1-positive cells were measured by the immunocytochemical analysis as described in the Materials and Methods. Dasatinib alone and dasatinib with Eto significantly decreased the proportion of ALDH1-positive cells in MDA-MB-231 cells. *P < 0.05.

**Figure 11:**
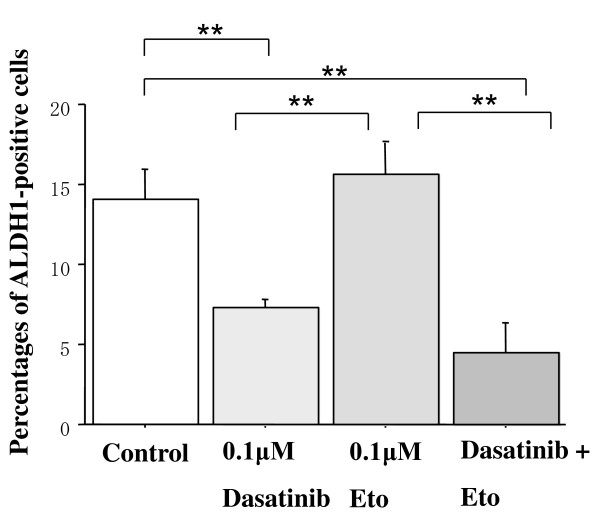
**Effects of dasatinib and/or Eto on the proportion of ALDH1-positive cells in MDA-MB-157 cells**. The cells were treated with 0.1 *μ*M Eto and/or 0.1 *μ*M dasatinib for two days, and ALDH1-positive cells were measured by the immunocytochemical analysis as described in the Materials and Methods. Dasatinib alone and dasatinib with Eto significantly decreased the proportion of ALDH1-positive cells in MDA-MB-157 cells. **P < 0.01.

**Figure 12:**
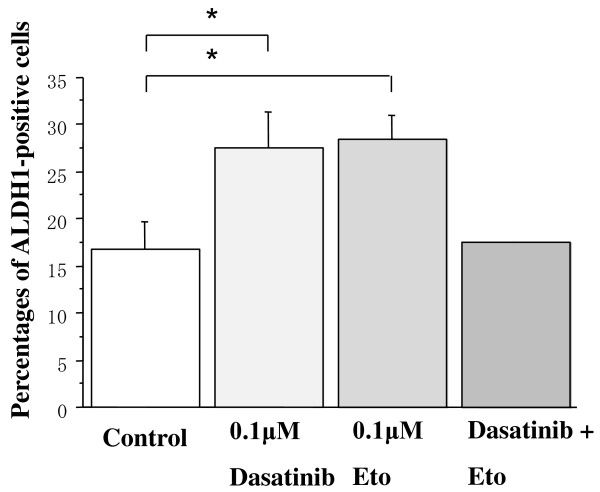
**Effects of dasatinib and/or Eto on the proportion of ALDH1-positive cells in BT-474 cells**. The cells were treated with 0.1 *μ*M Eto and/or 0.1 *μ*M dasatinib for two days, and ALDH1-positive cells were measured by the immunocytochemical analysis as described in the Materials and Methods. Dasatinib slightly increased the proportion of ALDH1-positive cells in BT-474 cells. *P < 0.05.

Dasatinib treatment (0.1 or 1.0 *μ*M) significantly decreased the proportion of ALDH1-positive cells in the cell lines of the basal B subtype (Figures 10 and 11). In contrast, it increased the proportion in the BT-474 cell line (Figure 12). Eto treatment (0.1 or 1.0 *μ*M) did not significantly decrease the proportion of ALDH1-positive cells in any of the cell lines tested. A combined treatment of dasatinib with Eto also significantly decreased the proportion of ALDH1-positive cells in the cell lines of the basal B subtype (Figures 10 and 11). The proportion of ALDH1-positive cells was 13.0 ± 1.4% for the control, 7.0 ± 1.6% for 0.1 μM dasatinib, 11.7 ± 0.1% for 0.1 μM Eto, and 5.2 ± 1.6% for combined treatment in MDA-MB-231 cells. The differences were significant among the control vs. dasatinib alone (P = 0.03) and Eto alone vs. combined treatment (P = 0.03). The proportion of ALDH1-positive cells was 14.1 ± 1.8% for the control, 7.3 ± 0.5% for 0.1 μM dasatinib, 15.6 ± 1.8% for 0.1 μM Eto, and 4.5 ± 1.9% for combined treatment in MDA-MB-157 cells. The differences were significant among the control vs. dasatinib alone (P = 0.01) and Eto alone vs. combined treatment (P < 0.01). No difference was observed between dasatinib alone and combined treatment in either cell line. These findings suggest that Eto did not enhance the decreased proportion of ALDH1-positive cells by dasatinib alone in these cell lines.

**Figure 13:**
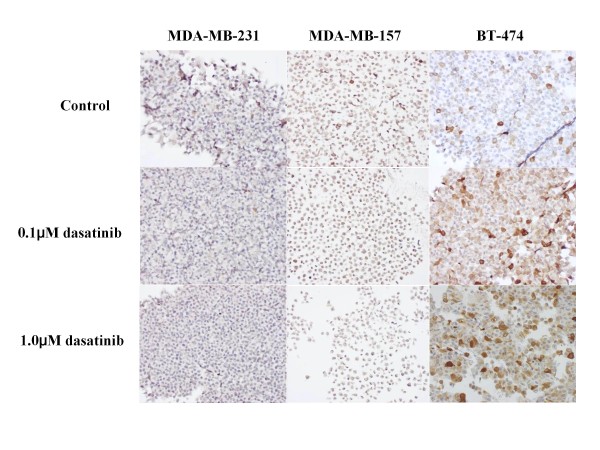
**Effects of dasatinib on the proportion of ALDH1-positive cells in breast cancer cells**. The immunocytochemical analyses demonstrated a decrease in the proportion of ALDH1-positive cells by dasatinib in MDA-MB-231 and MDA-MB-157 cells but an increase in the proportion of ALDH1-positive cells by dasatinib in BT-474 cells.

**Figure 14:**
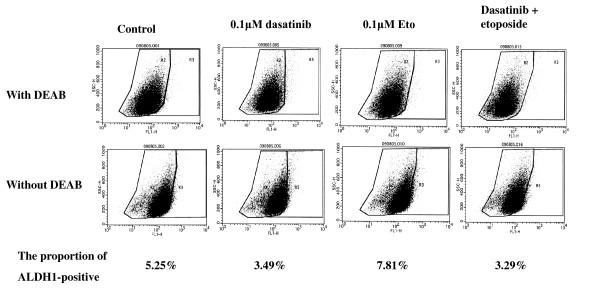
**Effects of dasatinib and/or Eto on the proportion of ALDH1-positive cells in breast cancer cells**. The Aldefluor assay demonstrated that dasatinib alone and dasatinib with Eto decreased the proportion of ALDH1-positive cells in MDA-MB-231 cells.

**Figure 15:**
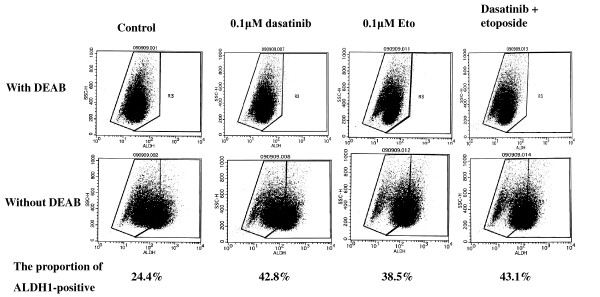
**Effects of dasatinib and/or Eto on the proportion of ALDH1-positive cells in breast cancer cells**. The Aldefluor assay demonstrated that dasatinib alone and dasatinib with Eto increased the proportion of ALDH1-positive cells in BT-474 cells.

Representative results demonstrated by the immunocytochemistry and Aldefluor assays are shown in Figures 13, 14 and 15.

## Discussion

To develop novel treatment strategies against triple negative/basal-like breast cancer, molecular targeting agents such as inhibitors of Src, poly(ADP-ribose) polymerase (PARP) 1, HER1 or the vascular endothelial growth factor (VEGF) signaling pathway have been tested in preclinical and clinical studies [4]. Dasatinib is an orally active small molecule inhibitor of both the Src and Abl, and was approved for the treatment of imatinib refractory chronic myelogenous leukemia and *bcr-abl *positive acute lymphoblastic leukemia [20]. Additionally, recent preclinical studies have indicated significant antitumor activity of dasatinib in breast cancer cells of the basal-like/triple negative subtype *in vitro *[5,6]. Other preclinical studies have also supported the role of Src inhibition with dasatinib in head and neck cancer, pancreatic cancer, lung cancer and osteosarcoma models [21-24]. Very recently, a preliminary study has shown that dasatinib monotherapy shows substantial antitumor activity in heavily-pretreated patients with triple negative metastatic breast cancer [25].

To further clarify the potential clinical role of the Src inhibitor dasatinib in breast cancer, we examined the *in vitro *effects of dasatinib using a panel of human breast cancer cell lines of four different subtypes (Additional file 1). Results of the present study have demonstrated that dasatinib effectively inhibited phosphorylation of c-Src in all cell lines tested (Figures 1, 2 and 3), and preferentially inhibited the growth of breast cancer cells of the basal B subtype (Additional file 2). The IC_50_s of dasatinib in the basal B breast cancer cell lines were approximately 4-6 times lower than the trough concentration (0.6 *μ*M) of dasatinib in humans [6]. These findings coincide with those reported by two independent groups [5,6].

To test a hypothesis that dasatinib may enhance antitumor activity of chemotherapeutic agents, we first examined the growth inhibitory effects of seven anti-cancer agents commonly used for the treatment of breast cancer. According to the IC_50 _for each agent, breast cancer cells of the basal B subtype seemed to be more sensitive to DNA-damaging agents such as Eto, Dox, Cis, Carb and SN38 than those of other subtypes, in particular, the luminal A and luminal B subtypes (Additional file 2). These findings may be partly explained by the facts that a loss of BRCA1 expression is frequently observed in breast cancer cells of the basal B subtype, and BRCA1 dysfunction enhances antitumor activity of DNA-damaging agents in breast cancer cells. Actually, BRCA1 expression levels measured by the immunocytochemical assay were significantly lower in three basal-like breast cancer cell lines (Additional file 1).

One of the DNA-damaging agents, Eto has long been used in the treatment of various malignancies such as lung cancer [26]. Recent clinical studies have also demonstrated that oral Eto in combination with intravenous Cis has a significant antitumor effect on metastatic breast cancer [27]. Since both dasatinib and Eto can be administered orally, breast cancer patients could be treated with these agents without intravenous injections. These findings prompted us to investigate a combined antitumor activity of dasatinib with Eto in breast cancer cells of the basal B subtype. Dasatinib (0.1 *μ*M) additively enhanced antitumor activity (Figures 4, 5, 6 and 7).

To elucidate the mechanism of action of combined treatment with dasatinib and Eto, the effects of single or combined treatments on cell cycle progression and induction of apoptosis were examined. Unexpectedly, Eto did not show any additive effect to the treatment with dasatinib alone on G1-S cell cycle retardation and increased apoptosis (Figures 8 and 9). In addition, the immunocytochemical and Aldefluor assay showed that Eto did not enhance the decreased proportion of ALDH1-positive cells by dasatinib alone (Figures 10, 11, 12, 13, 14 and 15). These findings suggest that the additive antitumor effect with dasatinib and Eto is unlikely to be due to enhanced cell cycle retardation, increased apoptosis or decreased proportion of ALDH1-positive, putative cancer stem cells. Further studies are needed to elucidate the mechanism of action responsible for possible additive antitumor activity with dasatinib and a chemotherapeutic agent. Otherwise, similar additive antitumor interactions were observed in the combinations of dasatinib with Pac and SN38 (Additional files 4, 5, 6 and 7). Combined administrations of dasatinib with chemotherapeutic agents may be more useful for the treatment of breast cancers of the basal B subtype.

Recent preclinical studies have suggested that a small component of tumor cells, so-called cancer stem cells play critical roles in the development, progression, metastasis and resistance to chemotherapy and radiotherapy in malignancies [28]. Interestingly, it was reported that a dual HER1/HER2 inhibitor lapatinib significantly reduced the proportion of CD44+/CD24- breast cancer cells, putative stem cells, and also reduced the number of mammosphere-forming activity associated with a significant antitumor activity in HER2-positive breast cancers in the neoadjuvant setting [13]. In contrast, cytotoxic chemotherapies including Dox increased the proportion of CD44/CD24- breast cancer cells and mammosphere-forming activity associated with a significant antitumor activity in HER2-negative breast cancers [13]. Notably, a very recent study has shown that cytotoxic chemotherapies including an anthracycline and taxane increased the proportion of ALDH1-positive breast cancer cells but not that of CD44+/CD24- breast cancer cells [29]. Additionally, another preclinical study has suggested that anti-HER2 monoclonal antibody trastuzumab effectively decreases the proportion of putative breast cancer stem cells in HER2-positive breast cancer cells [12]. More recently, an anti-diabetes mellitus agent metformin and 8-quinolinol have been reported to effectively target breast cancer stem cells and synergistically inhibit tumor growth together with the chemotherapeutic agents Dox and Pac, respectively [30]. These emerging preclinical data suggest that a combined use of anti-stem cell agents with chemotherapeutic agents might provide a longer tumor regression as well as cure for patients with malignancies.

To investigate the effects of dasatinib and Eto on breast cancer stem cells, the proportion of ALDH1-positive cells was examined before and after the treatments with these agents using immunocytochemistry and Aldefluor assay. Interestingly, dasatinib significantly decreased the proportion of ALDH1-positive cells in breast cancer cell lines of the basal B subtype, which were highly sensitive to dasatinib. In contrast, dasatinib significantly increased the proportion of ALDH1-positive cells in the BT-474 cell line, which was categorized as the luminal B subtype and insensitive to dasatinib. A cytotoxic agent Eto did not significantly decrease the proportion of ALDH1-positive cells in breast cancer cell lines of either luminal B or basal B subtypes (Figures 10, 11 and 12). These findings strongly suggest for the first time that the Src inhibitor dasatinib preferentially decreased the proportion of ALDH1-positive, putative breast cancer stem cells in breast cancer cells of the basal B subtype. However, mechanisms of action responsible for the anti-stem cell activity of dasatinib still remain to be elucidated. A recent preclinical study indicated that ablation of focal adhesion kinase (FAK) reduces the proportion of cancer stem cells in either an *in vitro *or *in vivo *model [31]. Because FAK is known to interact with the Src family members and its activation is reported to be suppressed by dasatinib, the inhibitory effect of dasatinib on the FAK signaling pathway might explain the anti-stem cell activity of dasatinib. Further studies are clearly needed to elucidate the mechanism of action of dasatinib responsible for its anti-stem cell activity.

## Conclusions

To the best of our knowledge, the present study has offered clear evidence that the Src inhibitor dasatinib preferentially inhibited the growth of breast cancer cells of the basal B subtype associated with a G1-S cell cycle blockade and a reduction in the proportion of ALDH1-positive, putative breast cancer stem cells. In addition, dasatinib significantly enhanced an anti-tumor activity of one of DNA-damaging agents Eto in breast cancer cells of the basal B subtype. These strong preclinical findings warrant clinical trials using dasatinib and Eto in patients with breast cancer of the basal B subtype.

## Competing interests

The authors declare that they have no competing interests.

## Authors' contributions

JK, NK, TM, YK, and MW made substantial contributions to the conception and design of the study, acquisition of data, and analysis and interpretation of the data. JK, NK, TM, and HS were involved in drafting the manuscript or revising it. All authors read and approved the final manuscript.

## Pre-publication history

The pre-publication history for this paper can be accessed here:

http://www.biomedcentral.com/1471-2407/10/568/prepub

## Supplementary Material

Additional file 1 **Table 1**. Biological characteristics of breast cancer cell lines Click here for file

Additional file 2 **Table 2**. The 50% growth inhibitory concentrations of dasatinib or chemotherapeutic agents in breast cancer cell lines Click here for file

Additional file 3 **Table 3**. Proportion of breast cancer stem cells measured by immunocytochemistry and Aldefluor assay Click here for file

Additional file 4 **Supplementary Figure 1**. Additive antitumor activity of dasatinib and SN38 in MDA-MB-231 cells. Click here for file

Additional file 5 **Supplementary Figure 2**. Additive antitumor activity of dasatinib and SN38 in MDA-MB-157 cells. Click here for file

Additional file 6 **Supplementary Figure 3**. Additive antitumor activity of dasatinib and Pac in MDA-MB-231 cells. Click here for file

Additional file 7 **Supplementary Figure 4**. Additive antitumor activity of dasatinib and Pac in MDA-MB-157 cells. Click here for file
