# Supplementary figures and images for: Preferential antitumor effect of the Src inhibitor dasatinib associated with a decreased proportion of aldehyde dehydrogenase 1-positive cells in breast cancer cells of the basal B subtype
Source: BMC Cancer. 2010 Oct 20;10:568. doi: 10.1186/1471-2407-10-568 (PMC2967550; doi:10.1186/1471-2407-10-568)

## Slide 1
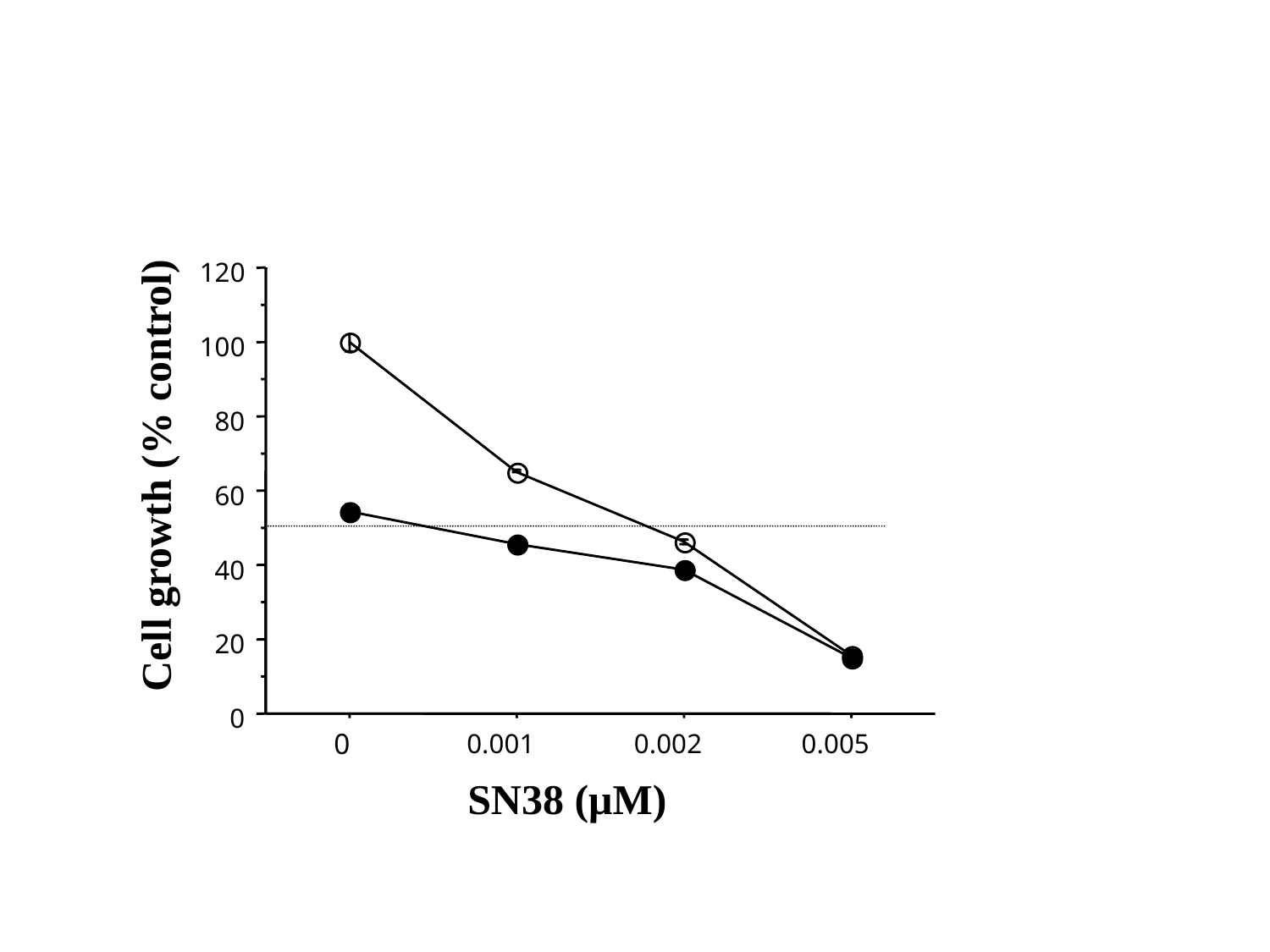

120
100
80
Cell growth (% control)
60
40
20
0
0
0.001
0.002
0.005
SN38 (μM)

Supplement: Additional file 4 — Supplementary Figure 1. Additive antitumor activity of dasatinib and SN38 in MDA-MB-231 cells. [file 1471-2407-10-568-S4.PPT]

## Slide 1
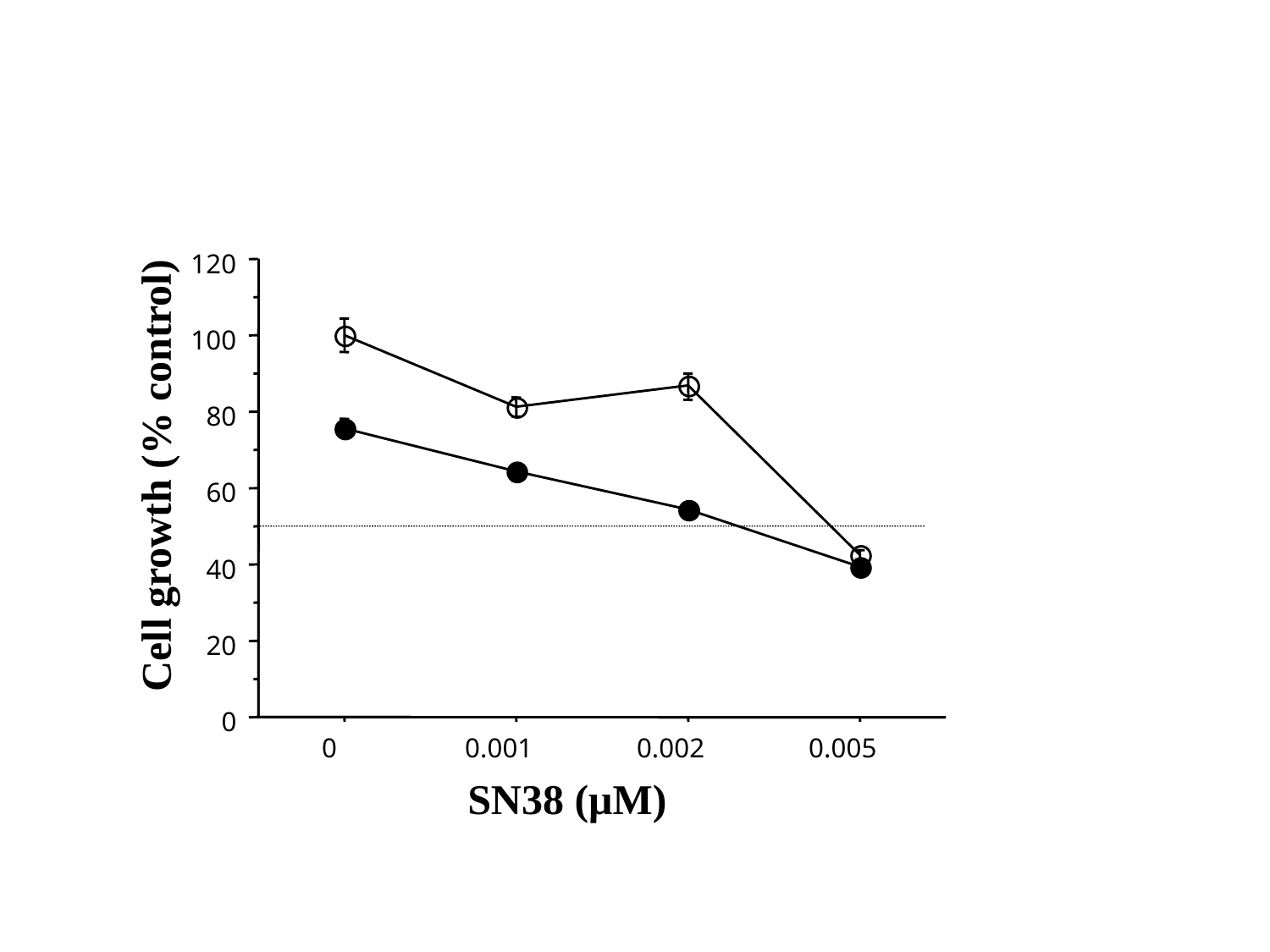

120
100
80
Cell growth (% control)
60
40
20
0
0
0.001
0.002
0.005
SN38 (μM)

Supplement: Additional file 5 — Supplementary Figure 2. Additive antitumor activity of dasatinib and SN38 in MDA-MB-157 cells. [file 1471-2407-10-568-S5.PPT]

## Slide 1
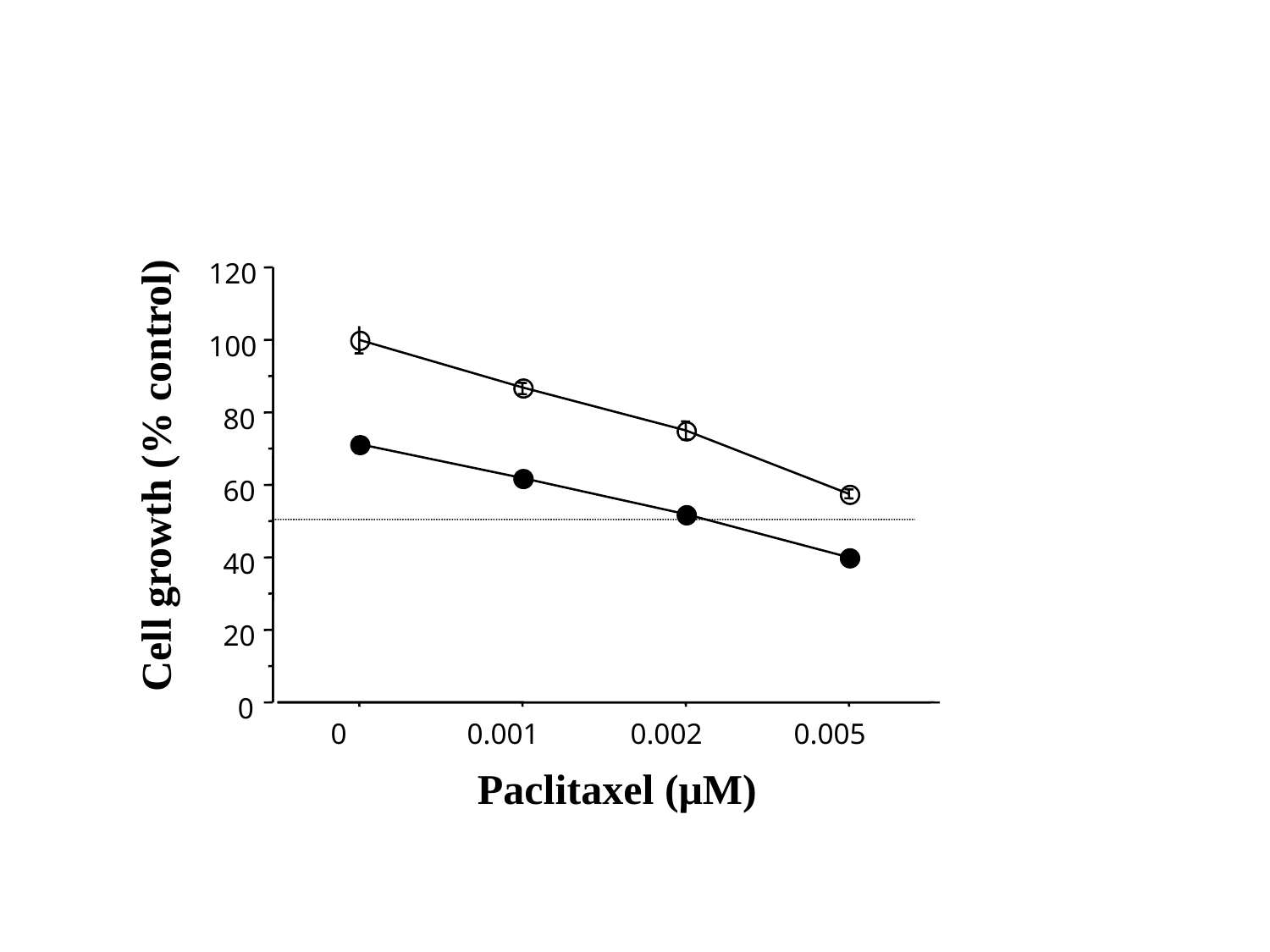

120
100
80
Cell growth (% control)
60
40
20
0
0
0.001
0.002
0.005
Paclitaxel (μM)

Supplement: Additional file 6 — Supplementary Figure 3. Additive antitumor activity of dasatinib and Pac in MDA-MB-231 cells. [file 1471-2407-10-568-S6.PPT]

## Slide 1
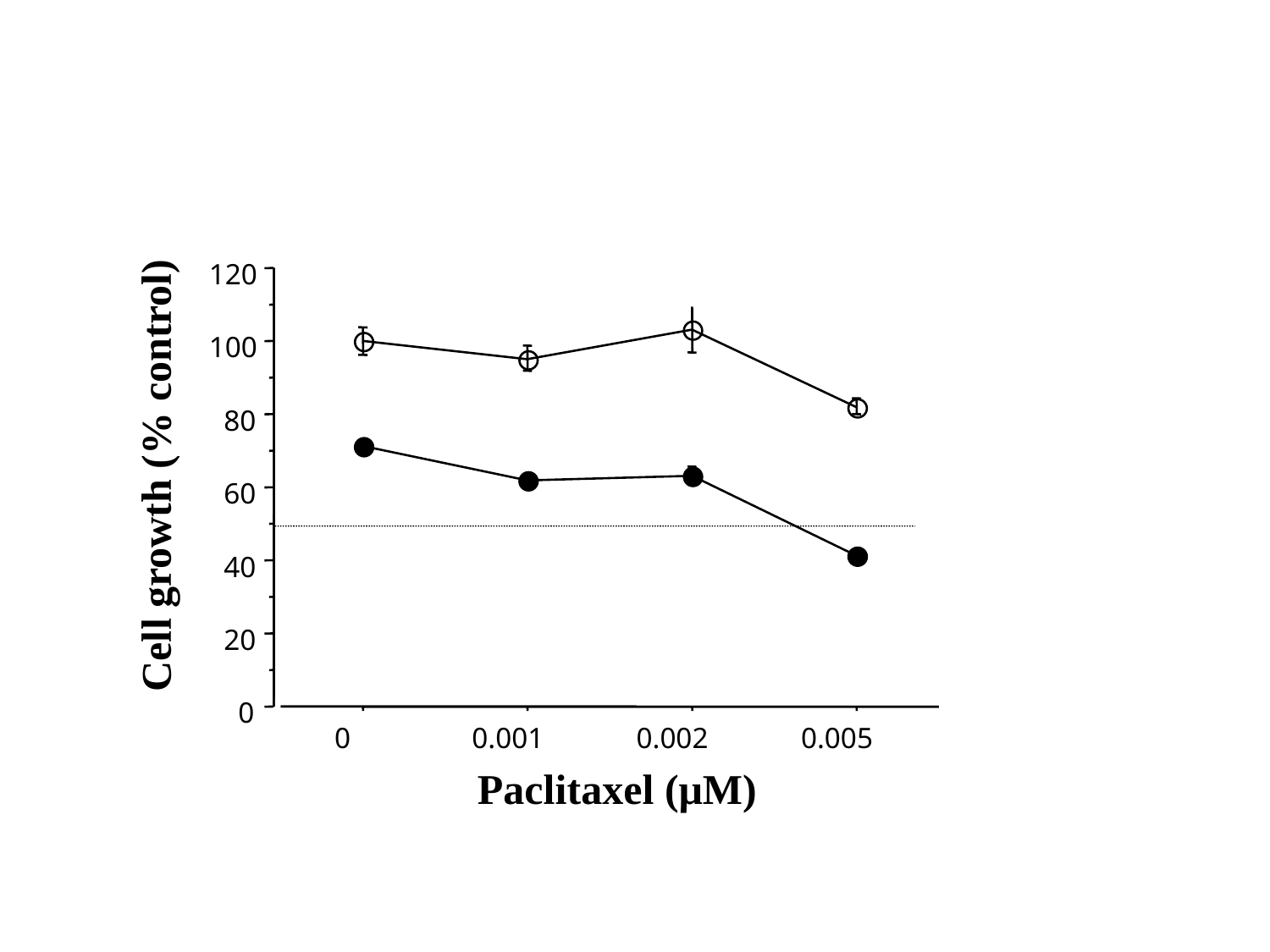

120
100
80
Cell growth (% control)
60
40
20
0
0
0.001
0.002
0.005
Paclitaxel (μM)

Supplement: Additional file 7 — Supplementary Figure 4. Additive antitumor activity of dasatinib and Pac in MDA-MB-157 cells. [file 1471-2407-10-568-S7.PPT]
